# The Interplay of SMAD4 and EMT in Oral Squamous Cell Carcinoma

**DOI:** 10.3390/cancers17111761

**Published:** 2025-05-23

**Authors:** Shiraz Mozalbat, Aysar Nashef, Naseem Maalouf, Murad Abdol-Elraziq, Imad Abu El-naaj, Hagar Tadmor, Yasmin Ghantous

**Affiliations:** 1 Molecular Biology of Oral Cancer Laboratory, Tzafon Medical Center, Azrieli Faculty of Medicine, Bar-Ilan University, Safed 1311502, Israel; shirazmzlbat@gmail.com (S.M.); hagart9@gmail.com (H.T.); 2 Department of Oral and Maxillofacial Surgery, Tzafon Medical Center, Faculty of Medicine, Bar-Ilan University, Ramat Gan 5290002, Israel; dr.aysarn@gmail.com (A.N.); naseem.maalouf@gmail.com (N.M.); muradabdol@gmail.com (M.A.-E.); iabu@tzmc.gov.il (I.A.E.-n.)

**Keywords:** oral squamous cell carcinoma (OSCC), epithelial–mesenchymal transition (EMT), lymph node (LN), biomarkers, Smad4

## Abstract

**Background:** Oral squamous cell carcinoma (OSCC) is a prevalent malignancy with a poor prognosis. Surgical removal of the primary tumor and regional lymph nodes (LNs) remains the fundamental treatment for OSCC, although 40% of patients are negative for LN metastasis. The epithelial–mesenchymal transition (EMT) plays a crucial role in OSCC progression by enabling epithelial cells to acquire mesenchymal traits, thereby facilitating migration and metastasis. Smad4, a tumor suppressor protein, is known to mediate EMT and is associated with poor prognosis and metastasis; however, its precise pro-metastatic role in OSCC via EMT remains unclear. **Aims:** We hypothesize that EMT and Smad4 could serve as practical diagnostic tools for personalized OSCC treatment. **Methods:** In this study, we analyzed 23 OSCC samples from Tzafon Medical Center, comparing the expression of Smad4 and EMT markers with clinical and histopathological data. Additionally, an OSCC cell model with and without Smad4 mutation was used to investigate tumor phenotypes, including proliferation and invasion, in relation to EMT markers. **Results and Conclusion:** Our findings reveal a strong correlation between EMT markers, Smad4 expression, and OSCC pathological staging, with the cell model further confirming the link between Smad4 and EMT markers. The combined influence of Smad4 and EMT markers on OSCC progression highlights their potential as diagnostic tools and as guides for personalized treatment strategies.

## Simple Summary

This study explores the complex relationship between SMAD4 and epithelial–mesenchymal transition (EMT) in oral squamous cell carcinoma (OSCC). By analyzing human tissue samples and cellular models, we found that higher SMAD4 expression correlates with early-stage OSCC and epithelial markers, while decreased SMAD4 is associated with increased mesenchymal markers and tumor invasiveness. Our findings suggest that SMAD4 plays a protective role against EMT-driven progression. These insights highlight the potential of SMAD4 and EMT markers as diagnostic tools and targets for personalized treatment strategies in OSCC.

## 1. Introduction

Head and neck squamous cell carcinoma (HNSCC) is the sixth most common cancer worldwide, with oral squamous cell carcinoma (OSCC) accounting for over 90% of HNSCC cases. OSCC is driven by genetic and environmental factors [1], exhibiting a high recurrence rate and a poorer prognosis, with a 5-year survival rate of approximately 50% [2]. Accurate staging of OSCC is critical for treatment planning and prognosis, commonly based on the TNM classification system. The standard treatment involves the surgical removal of the primary tumor and cervical lymph node (LN) dissection [3]. According to the 8th edition of the AJCC cancer staging manual, nearly 90% of OSCC patients undergo neck dissection [4]. However, pathology results often show negative findings for LN metastasis in the majority of patients staged as T1/T2N0, making the necessity of neck dissection in early-stage cases a subject of debate [5].

Epithelial–mesenchymal transition (EMT) is a reversible, dynamic process whereby epithelial cells acquire mesenchymal traits, such as increased motility, invasiveness, and resistance to apoptosis [6]. EMT involves morphological changes, loss of cell–cell adhesion, and alterations in epithelial and mesenchymal marker expression [7]. The expression of EMT markers plays a significant role in cancer progression and is associated with aggressive tumor behavior and poor prognosis. A hallmark of EMT is the “E/N cadherin switch”, characterized by the loss of E-cadherin and the overexpression of N-cadherin, which promotes invasion and metastasis [7]. Elevated N-cadherin levels have been linked to increased invasiveness and metastasis in various cancers, including breast, prostate, pancreatic, and OSCC [8]. Similarly, upregulated during EMT, Vimentin is associated with enhanced metastasis and reduced overall survival [9].

EMT is primarily triggered by signaling pathways such as TGF-β, Wnt, and Notch, which activate transcription factors that suppress E-cadherin and promote mesenchymal traits [6]. Conversely, the reverse process, mesenchymal–epithelial transition (MET), involves the re-establishment of cell–cell adhesion and epithelial characteristics, facilitating secondary tumor formation at metastatic sites [6].

Smad4, a central mediator of the TGF-β pathway, functions as a tumor suppressor in OSCC by inhibiting proliferation, inducing apoptosis, and preventing metastasis [10]. Reduced Smad4 levels are associated with increased tumor aggressiveness, advanced stages, and poorer prognosis [10,11]. Evidence suggests that Smad4 influences EMT, with its loss or mutation disrupting TGF-β signaling and abnormal EMT activation in cancer [11]. The disruption of Smad4–receptor interactions can inhibit EMT, preserving epithelial traits such as adhesion, polarity, and differentiation, thereby suppressing invasion, metastasis, and therapy resistance [11]. However, the precise role of Smad4 in OSCC progression and its mechanisms in EMT and metastasis remain incompletely understood.

This study aims to evaluate the expression patterns of Smad4 and EMT markers in human OSCC tissues and cell models, elucidating their roles in tumor progression and metastasis.

## 2. Materials and Methods

### 2.1. Human Tissue Samples

Oral cancer patients were recruited from the Oral and Maxillofacial Department of Tzafon Medical Center, following approval from the Helsinki Committee of Tzafon Medical Center and the Israel Ministry of Health (Helsinki Committee No. 0122-18-POR). Patients diagnosed with OSCC (confirmed by previous pathological analysis) and eligible for neck dissection were included. Clinical and demographic data—such as gender, age, smoking status, alcohol use, chronic diseases, and tumor characteristics—were collected and documented. Cancerous and healthy tissues were obtained in accordance with ethical approval and stored for histological and genetic analyses.

### 2.2. OSCC Cell Lines and Culture Conditions

OSCC cell lines (ATCC, Manassas, VA, USA) were used as models with or without mutations in the *Smad4* gene (Cal27 CRL-2095 and SCC25 CRL-1628, respectively). Cells were cultured in media recommended by the manufacturer, supplemented with 10–20% FBS and 1% penicillin/streptomycin, at 37 °C in a 5% CO_2_ atmosphere. Genetic manipulations, such as transfections, were performed based on the *Smad4* status of the cells. EMT marker expression and Smad4 levels were assessed before and after transfection to evaluate effects on tumor phenotypes, including proliferation and invasion.

### 2.3. Genetic Manipulation of Smad4: Cell Co-Transfection

Cells in the exponential growth phase were seeded into six-well plates and cultured at 37 °C with 5% CO_2_. The following day, co-transfection was performed using Lipofectamine™ 2000 (Thermo Fisher Scientific, Waltham, MA, USA). For cells with intact *Smad4*, lentiviral shRNA and eGFP (Addgene, Watertown, MA, USA) were used to knock down *Smad4*. For *Smad4* mutant cells, overexpression plasmids for *Smad4* and eGFP were transfected. After 6 h, the medium was replaced, and transfected cells were verified visually via GFP fluorescence and selected with puromycin (Thermo Fisher Scientific, Waltham, MA, USA). At 24 h post selection, the medium was replaced with antibiotic-free medium. SCC25 cells with a Tet-on system received doxycycline (Sigma-Aldrich, St. Louis, MO, USA).

### 2.4. Hematoxylin and Eosin (H&E) Staining

Tumor tissues were fixed in 4% formaldehyde, paraffin-embedded, and sectioned at 4 μm thickness. Sections were deparaffinized at 80 °C, hydrated through graded alcohols (100%, 95%, 70%), and stained with Mayer’s hematoxylin (Leica, Teaneck, NJ, USA) and eosin (Leica, Teaneck, NJ, USA). After dehydration, slides were mounted with coverslips. Morphological analysis was performed using an AxioLab A1 microscope equipped with Axiocam 105 color digital camera (Carl Zeiss Microscopy GmbH, Germany) and ZEN software (Version 3.10.103.04000).

### 2.5. Immunohistochemistry (IHC) Staining

Deparaffinized tissue sections were rehydrated, incubated with hydrogen peroxide for antigen retrieval, and then incubated overnight at 4 °C with primary antibodies against E-cadherin, N-cadherin, Vimentin, and Smad4 (1:100, ab40772; 1:100, ab76011; 1:200, ab92547; 1:100, ab40759; all from Abcam, Cambridge, UK). Secondary detection was performed with HRP–polymer anti-rabbit antibodies (Nichirei, Dako, NE, USA). Sections were counterstained with hematoxylin, mounted with a quick-hardening medium, and visualized using DAB Plus Substrate (Thermo Fisher Scientific, Waltham, MA, USA). Images were captured with the AxioLab A1 microscope (Carl Zeiss Microscopy GmbH, Germany) and protein levels were quantified using ImageJ software (Version 1.38).

### 2.6. Molecular Analysis: Real-Time PCR

Total RNA was extracted from tissues and cells using the QIAGEN RNeasy Mini Kit (QIAGEN, Hilden, Germany). cDNA synthesis was performed with a High-Capacity cDNA Reverse Transcription Kit (QIAGEN). Quantitative RT-PCR was carried out on a Bio-Rad CFX384 system (Bio-Rad, Hercules, CA, USA) using SYBR Green Master Mix (Thermo Scientific). Expression levels were normalized to β-actin, and results were calculated using the 2^−ΔΔCt^ method. Primer sequences are listed in Table 1.

### 2.7. Immunofluorescence (ICC-F)

Immunofluorescence was used to confirm transfection efficiency and analyze EMT marker expression before and after *Smad4* modification. Cells were seeded into 24-well plates, fixed with 4% paraformaldehyde, subjected to antigen retrieval, and permeabilized. Non-specific binding was blocked with 2% BSA. Cells were incubated overnight at 4 °C with primary antibodies against Smad4 (1:100, ab230815), E-cadherin, N-cadherin, and Vimentin (1:500, ab231303; 1:200, ab19348; 1:1000, ab16700; all from Abcam). Secondary antibodies used were goat anti-rabbit AlexaFluor 488 (ab150061, abcam, UK) and goat anti-mouse AlexaFluor 594 (ab150120, abcam, UK). Nuclei were stained with DAPI (H-1200, Vectashield, Switzerland), and images were captured using a fluorescence microscope (EVOS M5000, Thermo Fisher Scientific) at 200× magnification.

#### 2.7.1. Functional Analysis: Wound-Healing Assay

Cells were seeded into 96-well plates and incubated for 48 h. A uniform scratch was created using a 96-pin device to generate consistent wounds across wells. Wound closure was monitored every two hours for 48 h using the IncuCyte^®^ SX5 imaging system (Sartorius, Göttingen, Germany). Migration rates were quantified using the IncuCyte^®^ Scratch Wound Analysis software (9600-0012, Sartorius, Germany).

#### 2.7.2. Functional Analysis: XTT Assay

SCC25 and Cal27 cells, before and after co-transfection, were seeded into 96-well plates and incubated for 24 h. Cell viability and proliferation were assessed using the XTT assay (Roche, Mannheim, Germany). Absorbance was measured at 450–630 nm using a microplate reader (Thermo Fisher Scientific) at 0, 6, and 24 h.

### 2.8. Statistical Analysis

Data are expressed as mean ± standard deviation. Statistical comparisons between groups were performed using Student’s *t*-test or ANOVA, with an analysis conducted via GraphPad Prism 5.00 (GraphPad Software, La Jolla, CA, USA). A *p*-value < 0.05 was considered statistically significant. All experiments were performed at least three times.

## 3. Results

### 3.1. Demographic and Clinical Characteristics of the Study Population

A total of 23 patients treated at the Department of Oral and Maxillofacial Surgery at Tzafon Medical Center were recruited after meeting the inclusion criteria. The clinical and demographic data are summarized in Table 2. All participants were diagnosed with OSCC based on pathology reports. Of these, 78% underwent selective neck dissection; among them, only 22% had confirmed lymph node metastasis. The mean age at diagnosis was 62 years. The cohort was composed of 52% females, and 34% reported tobacco use. The most common tumor site was the oral tongue (43%), followed by the lower and upper alveolus (30%).

### 3.2. Morphological Features of OSCC Tissues

#### Morphological Features of Oral Tissues with Vascular Invasion and Dysplastic Epithelium

**Figure 1:**
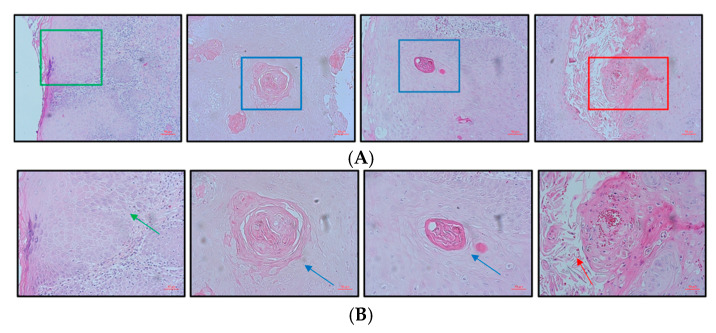
H&E staining of OSCC tissue samples showing morphological alterations: the invasion and severe dysplasia of surface epithelium (green arrows), central keratinization (blue arrows), and local inflammatory response (red arrows). (**A**) Representative low-magnification image (×4). (**B**) Higher magnification of the enlarged area (×20).

Histological examination using H&E staining revealed morphological alterations in oral tissue sections, including dysplasia, invasion, and central keratinization with abnormal squamous cells (Figure 1A, ×4; Figure 1B, ×20). These features indicate invasive tumor growth and epithelial dysplasia.

### 3.3. Expression of Smad4 and EMT Markers in Tumor Tissues

**Figure 2:**
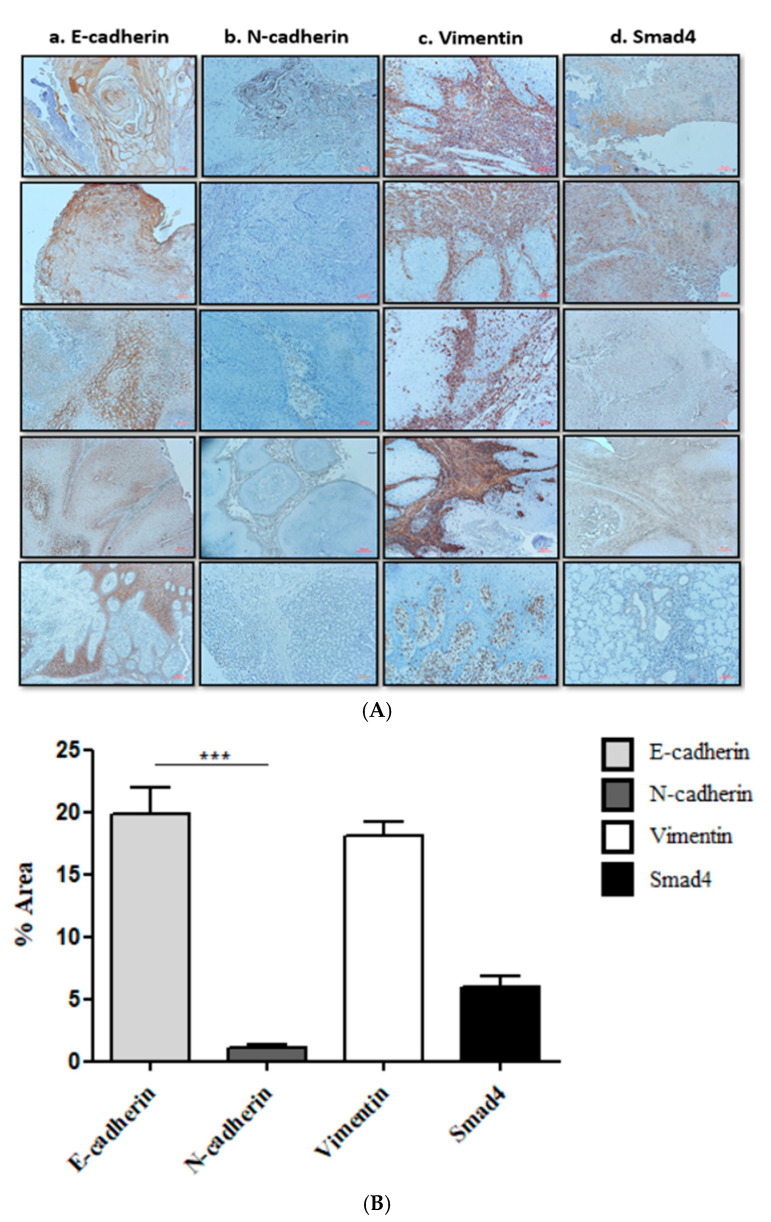
Immunohistochemical staining of EMT markers and Smad4 in OSCC tissues. (**A**) Representative images showing E-cadherin, N-cadherin, Vimentin, and Smad4 expression at ×20 magnification. (**B**) Quantitative analysis of staining intensity (% positive cells) in 23 samples.

E-cadherin showed relatively high membrane expression (Figure 2A(a)), whereas N-cadherin was less expressed (Figure 2A(b)). The “E/N cadherin switch”, a hallmark of EMT progression, was more evident in early-stage OSCC (Stages I and II), where E-cadherin expression was higher relative to N-cadherin. Vimentin was localized mainly in the cytoplasm (Figure 2A(c)), and nuclear Smad4 was predominantly located within cell nuclei (Figure 2A(d)). Both EMT markers and Smad4 showed higher expression levels in early-stage OSCC. Quantitative analysis (Figure 2B) confirmed these observations: E-cadherin expression was significantly higher (19.8 ± 9.05%) compared to N-cadherin (1.1 ± 1.5%; paired *t*-test, *** *p* < 0.0001), with elevated Vimentin and Smad4 expressions as well.

### 3.4. Gene Expression of EMT Markers and Smad4 in Tumor Versus Healthy Tissues

**Figure 3:**
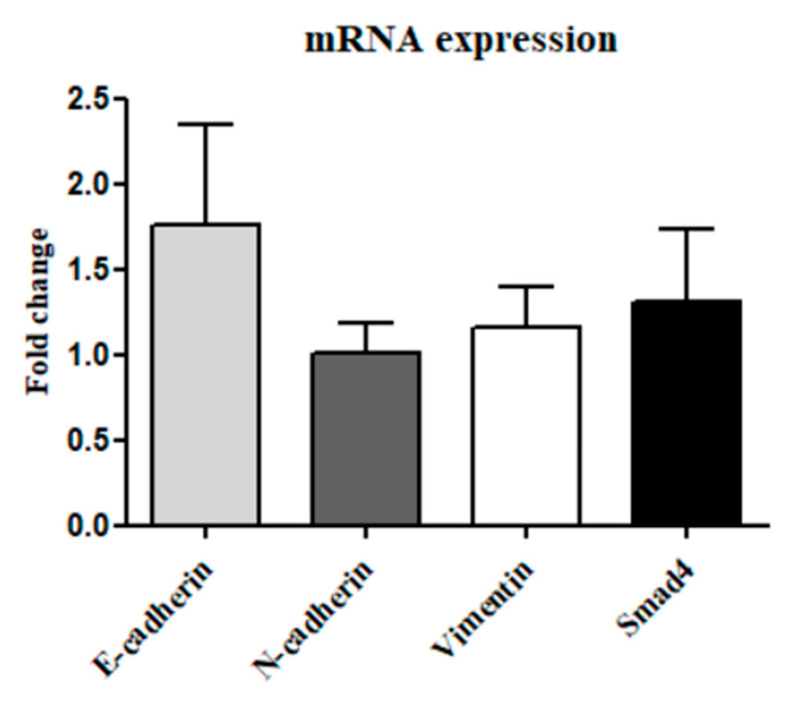
Fold change in gene expression of EMT markers and Smad4 in tumor versus healthy tissues (n = 23).

RT-PCR analysis evaluated the mRNA levels of EMT markers and Smad4 in tumor and healthy tissues (n = 23). The results showed increased levels of E-cadherin and Smad4 compared to N-cadherin and Vimentin; however, statistical analysis via paired *t*-test revealed no significant differences between E- and N-cadherin or among EMT markers and Smad4 between healthy and tumor tissues (Figure 3).

E-cadherin and Smad4 exhibited higher mean expression levels, but differences were not statistically significant.

### 3.5. In Vitro Modulation of Smad4 and EMT Markers

**Figure 4:**
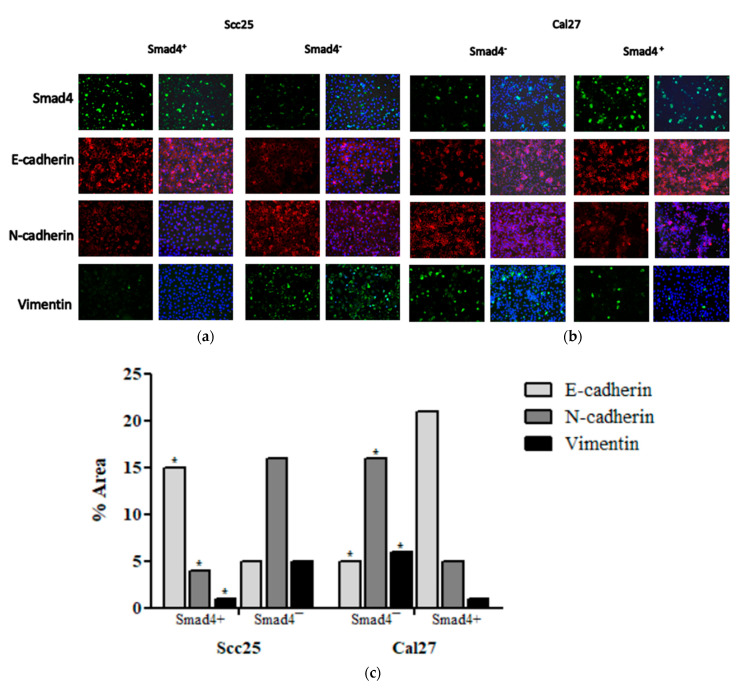
An immunofluorescence analysis of EMT markers and Smad4 expression in OSCC cell line with/without *Smad4* mutation: (**a**,**b**) demonstrate OSCC cell line, which expresses differences between EMT markers and Smad4 staining in the Scc25/Cal27 pre- and post transfection (**c**). The quantification of EMT markers. The results represent the mean expression level of each gene. The Mann–Whitney test was performed to obtain the *p*-value indicated on the graph (* *p* = 0.0286). Scc25 *Smad4*- shows a significant increase in N-cadherin (16.2 ± 0.2%) and Vimentin (5.9 ± 0.03%), and a significant decrease in E-cadherin (5.06 ± 0.08%) compared to Scc25 pre-transfection levels of E-cadherin (15.7 ± 0.42%, Mann–Whitney test, * *p* = 0.0286), N-cadherin (4.5 ± 0.32%, Mann–Whitney test, * *p* = 0.0286), and Vimentin (0.16 ± 0.4%, Mann–Whitney test, * *p* = 0.0286). Cal27 *Smad4+* shows a significant increase in N-cadherin (5.3 ± 0.33%) and Vimentin (1.3 ± 0.14%) expression, and significantly high expression in the level of E-cadherin (20.9 ± 1.2%) compared to Cal27 *Smad4-* levels of E-cadherin (4.9 ± 0.52%, Mann–Whitney test, * *p* = 0.0286), N-cadherin (16.5 ± 0.23%, Mann–Whitney test, * *p* = 0.0286), and Vimentin (6.5 ± 0.18%, Mann–Whitney test, * *p* = 0.0286).

Immunofluorescence confirmed differences in EMT markers and Smad4 expression in cell lines before and after genetic modification. Scc25 cells with *Smad4* knockdown (*Smad4*-) showed increased N-cadherin (16.2 ± 0.2%) and Vimentin (5.9 ± 0.03%), and decreased E-cadherin (5.06 ± 0.08%) compared to pre-transfection levels (E-cadherin: 15.7 ± 0.42%; N-cadherin: 4.5 ± 0.32%; Vimentin: 0.16 ± 0.4%; Mann–Whitney test, * *p* = 0.0286). Conversely, Cal27 cells overexpressing *Smad4* (*Smad4*+) displayed increased E-cadherin (20.9 ± 1.2%) and decreased N-cadherin (5.3 ± 0.33%) and Vimentin (1.3 ± 0.14%) compared to Smad4- cells (E-cadherin: 4.9 ± 0.52%; N-cadherin: 16.5 ± 0.23%; Vimentin: 6.5 ± 0.18%; *p* < 0.05). See Figure 4.

**Figure 5:**
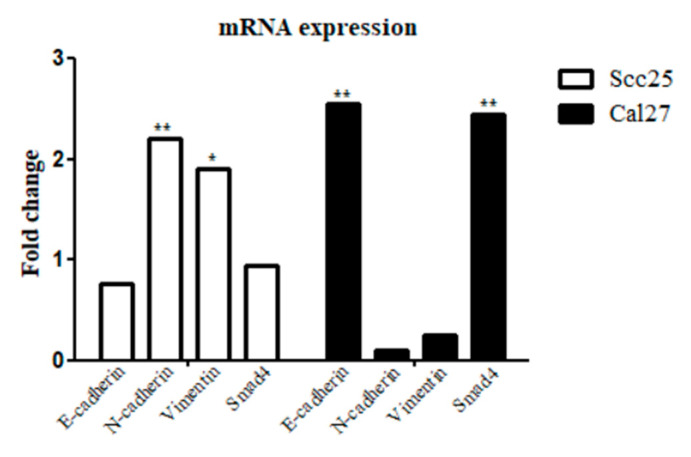
Fold-change alteration in pre- and post-transfection cells. Alteration in gene expression of EMT markers and Smad4 OSCC cells before and after Smad4 gene modification. Scc25 *Smad4*- compared to Scc25 *Smad4*+ shows a decrease in *Smad4* (0.94 ± 0.39, not significantly, unpaired *t*-test, *p* = 0.0647) and E-cadherin (0.76 ± 0.79, not significantly, unpaired *t*-test, *p* = 0.1499), and a significant increase in N-cadherin (2.2 ± 1.19, unpaired *t*-test, ** *p* = 0.0071) and Vimentin (1.9 ± 0.57, unpaired *t*-test, * *p* = 0.0181). Cal27 *Smad4+* compared to Cal27 *Smad4-* shows a significant increase in *Smad4* (2.45 ± 0.33, unpaired *t*-test, ** *p* = 0.0049) and E-cadherin (2.54 ± 0.75, unpaired *t*-test, ** *p* = 0.0019), and a low expression of N-cadherin (0.10 ± 0.75, not significantly, unpaired *t*-test, *p* = 0.7625) and Vimentin (0.25 ± 0.89, not significantly, unpaired *t*-test, *p* = 0.2380).

Correspondingly, RT-PCR analysis revealed that EMT marker expression changed according to the expression of Smad4: Smad4-positive cells exhibited higher E-cadherin and lower N-cadherin and Vimentin levels, whereas Smad4-depleted cells showed the opposite pattern (Figure 5).

### 3.6. Functional Assays Demonstrate Smad4’s Regulatory Role

#### 3.6.1. Wound-Healing Assays

**Figure 6:**
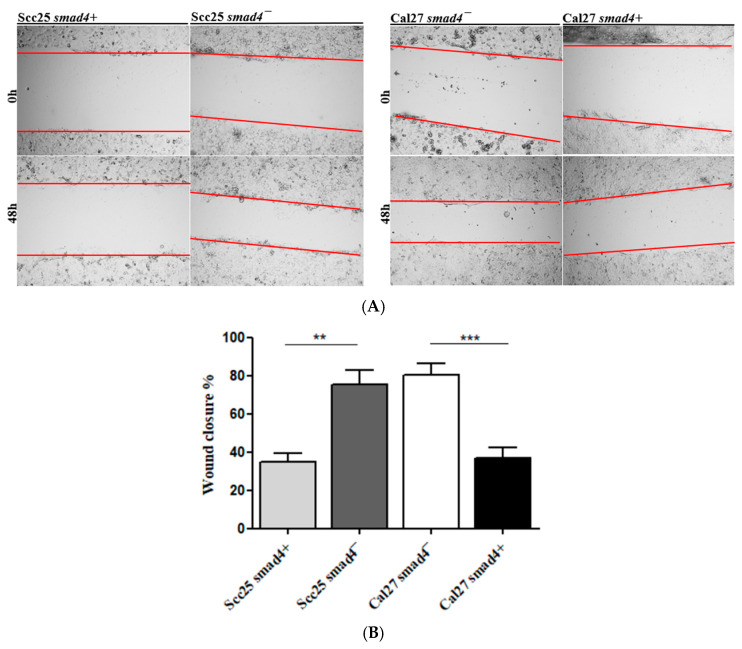
Tumor-like phenotype: wound-healing assay in OSCC cell lines with/without *Smad4* mutation (**A**) Representative images of wound closure at 0 h and 48 h (×10). (**B**) Quantification of wound closure (%): Scc25 *Smad4*- (81.2 ± 14, n = 12) demonstrated significantly increased wound closure compared to Scc25 *Smad4*+ (37.2 ± 12.5, Mann–Whitney test, ** *p* = 0.0011). Similarly, Cal27 *Smad4*+ (34.7 ± 10.7, n = 12) showed reduced wound closure compared to Cal27 *Smad4*- (83.4 ± 15.7, *** *p* = 0.0003).

The wound healing assay measured cell motility and migration by creating a scratch in the cell monolayer. Over 48 h, Scc25 cells with *Smad4* knockdown (*Smad4*-) showed significantly higher migration and motility (greater wound closure) compared to Scc25 cells with intact *Smad4* (*Smad4*+). Conversely, Cal27 cells overexpressing *Smad4* (*Smad4*+) exhibited lower wound-closure percentages compared to Cal27 cells with *Smad4* knocked down (*Smad4*-), as shown in Figure 6.

#### 3.6.2. XTT Proliferation Assays

**Figure 7:**
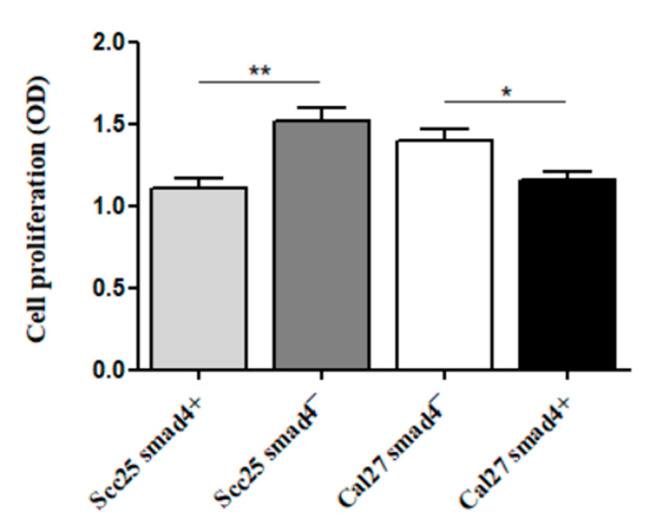
Tumor-like phenotypes in OSCC cell lines with/without Smad4 mutation: The quantification of proliferation via XTT assay. Data represent the mean ± SEM from three replicates. Scc25 Smad4- cells showed increased proliferation (0.73 ± 0.4, n = 10) compared to Scc25 Smad4+ (0.93 ± 0.6, ** *p* = 0.0012). Conversely, Cal27 Smad4+ cells exhibited reduced proliferation (0.77 ± 0.4, n = 10) compared to Cal27 Smad4- (1.0 ± 0.4, * *p* = 0.0142).

The XTT proliferation assay revealed that *Smad4* knockdown in Scc25 cells led to significantly increased proliferation (0.93 ± 0.6, n = 10) compared to Scc25 cells with intact *Smad4* (*Smad4*+). The same trend was observed in Cal27 cells, where *Smad4* overexpression resulted in decreased proliferation compared to *Smad4*-depleted cells (Figure 7). These results suggest that Smad4 negatively regulates cell proliferation in OSCC.

## 4. Discussion

Epithelial–mesenchymal transition (EMT) is a well-characterized mechanism in tumor progression and metastasis. During EMT, epithelial cells—characterized by tight cell-to-cell adhesion and basal polarity—lose these properties, gaining increased migratory and invasive capabilities. Alterations in EMT markers have been implicated in promoting metastasis across various cancers, including breast, lung, colorectal, and oral carcinomas [12,13,14].

Angadi et al. reported that morphological changes associated with EMT, including decreased E-cadherin and increased N-cadherin expression, are essential for OSCC cells to invade underlying connective tissue and metastasize to lymph nodes [15]. Kaur et al. (2013) and Gonzalez-Moles et al. (2014) highlighted the crucial role of E-cadherin interactions with catenins, which anchor cell-adhesion complexes to the actin cytoskeleton, facilitating cellular cohesion. The disruption of this complex promotes a shift to N-cadherin expression, correlating with increased invasiveness and high histological grade, particularly in advanced-stage disease [16,17,18].

Despite the established role of EMT, some studies have observed that lymph node metastasis (LNM) specimens can retain normal E-cadherin levels, suggesting that tumor cells may employ multiple survival strategies during metastasis, including the modulation of cell division rates while maintaining certain epithelial characteristics [19]. The pivotal role of EMT in tumor progression and dissemination underscores its potential as a predictive marker [6,20].

In this study, we examined the expression of EMT markers and Smad4 in human oral tissues to evaluate their diagnostic potential. Our findings indicated that early-stage OSCC tissues exhibit higher E-cadherin and Smad4 expression, along with lower N-cadherin and Vimentin levels, suggesting their utility as early indicators of tumor progression. To our knowledge, this is the first report demonstrating the combined use of EMT markers and Smad4 to assess OSCC progression in relation to pathological staging.

Smad4 is a key transcription factor in the TGF-β signaling pathway, which influences tumor growth and EMT. Several studies have shown that TGF-β induces EMT through Smad-dependent pathways, with Smad4 playing a critical role [11,21]. Intracellular Smad4 levels modulate the pathway’s activity: high levels inhibit proliferation and EMT, while low levels may permit EMT initiation and increased invasiveness [21].

Our cellular models confirmed this relationship. Smad4 knockdown in Scc25 cells led to increased N-cadherin and Vimentin expression, decreased E-cadherin, and enhanced migratory capacity, as demonstrated by wound-healing assays. Conversely, the overexpression of Smad4 in Cal27 cells suppressed EMT markers and reduced migration. These results reinforce Smad4’s role as a suppressor of EMT and tumor invasiveness.

The combination of protein- and gene-expression analyses, along with functional assays, underscores the importance of Smad4 in regulating EMT and tumor aggressiveness. Our findings suggest that assessing EMT markers alongside Smad4 could serve as a valuable diagnostic and prognostic tool, guiding personalized treatment strategies in OSCC.

While this study provides valuable insights into the relationship between Smad4 and EMT markers in OSCC, several limitations should be acknowledged. First, the sample size of 23 patients, although sufficient for preliminary analysis, may not fully capture the heterogeneity of OSCC across diverse populations, limiting the generalizability of the findings. Second, the cross-sectional design precludes establishing causality between Smad4 expression and EMT activation; longitudinal studies are necessary to confirm these relationships over time. Third, the in vitro models, while informative, cannot fully recapitulate the complex tumor microenvironment, including interactions with stromal and immune cells, which also influence EMT and tumor progression. Additionally, the molecular mechanisms underpinning Smad4’s regulatory effects on EMT require further elucidation, including downstream signaling pathways and post-transcriptional modifications. Future larger-scale, multi-center studies and in vivo investigations are needed to validate these findings and explore their clinical applicability.

## 5. Conclusions

This study demonstrates a strong association between Smad4 expression and EMT marker profiles in both human OSCC tissues and cell models. Specifically, early-stage tumors showed higher Smad4 and epithelial marker expression, with lower mesenchymal markers, correlating with less aggressive disease and the absence of metastasis. Conversely, the loss of Smad4 was associated with increased EMT marker expression and enhanced tumor-cell migration and proliferation.

## Figures and Tables

**Table 1:** The sequences of Smad4 and EMT markers’ primers.

Gene	Forward Primer (5′ > 3′)	Reverse Primer (5′ > 3′)
E-cadherin	TGCCCAGAAAATGAAAAAGG	GGATGACAGCGTGAGAGA
N-cadherin	GACAATGCCCCTCAAGTGTT	CCATTAAGCCGAGTGATGGT
Vimentin	CCCTCACCTGTGAAGTGGAT	TCCAGCAGCTTCCTGTAGGT
*Smad4*	ACCACCAAAACGGCCATCTTCAG	GGTCCACGTATCCATCAACAGTA
β-actin	GGACTTCGAGCAAGAGAT	AGCACTGTGTTGGCGTAC

**Table 2:** General demographic and clinical characteristics of the study population.

Characteristic	OSCC Patients (n = 23)
**Age (years)**	62 ± 13 years
**Gender** Female = F Male = M	F = 12 (52%) M = 11 (48%)
**Tobacco exposure** **Num. (%)**	8 (34%)
**Alcohol consumption (%)**	4 (15%)
**Primary tumor site (%)** Oral tongue Mouth floor Buccal Mucosa Lower + Upper alveolus Retromolar Lip	10 (43%) 2 (8%) 1 (4%) 7 (30%) 1 (4%) 2 (8%)
**T stage** T1 (%) T2 (%) T3 (%) T4 (%)	7 (30%) 9 (39%) 2 (8%) 3 (13%)
**N stage** N0 (%) N1 (%) N2 (%) N3 (%)	14 (78%) 2 (11%) 2 (11%) 1 (0.04%)
**M stage**	0
**TNM stage** Stage I (%) Stage II (%) Stage III (%) Stage IV (%)	10 (43%) 8 (34%) 1 (4%) 4 (17%)
**Neck dissection**	18 (78%) 5 (22%)

Pathological staging according to the American Joint Committee on Cancer (AJCC).

## Data Availability

The data presented in this study are available on request from the corresponding authors.

## Abbreviations

The following abbreviations are used in this manuscript

HNSCC	Head and neck squamous cell carcinoma
OSCC	Oral squamous cell carcinoma
LN	Lymph node
ND	Neck dissection
AJCC	American Joint Committee on Cancer
EMT	Epithelial–mesenchymal transition
MET	Mesenchymal–epithelial transition
E-cadherin	Epithelial cadherin
N-cadherin	Neural cadherin
TGF-β	Transforming growth factor β
PBS	Phosphate-buffered saline
HRP	Horseradish peroxidase
cDNA	Complementary DNA
H&E	Hematoxylin and Eosin
WH	Wound healing
RT-PCR	Real-time reverse transcription polymerase chain reaction
LNM	Lymph node metastasis
